# Following a potential epileptogenic insult, prolonged high rates of nonlinear dynamical regimes of intermittency type is the hallmark of epileptogenesis

**DOI:** 10.1038/srep31129

**Published:** 2016-08-04

**Authors:** Massimo Rizzi, Itai Weissberg, Dan Z. Milikovsky, Alon Friedman

**Affiliations:** 1 Department of Neuroscience, IRCCS - Istituto di Ricerche Farmacologiche Mario Negri, Via G. La Masa 19, 20156 Milan, Italy; 2 Departments of Physiology and Cell Biology, Cognitive and Brain Sciences, Zlotowski Center for Neuroscience, Ben-Gurion University of the Negev, Beer-Sheva 84105, Israel; 3 Department of Medical Neuroscience, Faculty of Medicine, Dalhousie University, Halifax, Nova Scotia B3H 4R2, Canada

## Abstract

The lack of a marker of epileptogenesis is an unmet medical need, not only from the clinical perspective but also from the point of view of the pre-clinical research. Indeed, the lack of this kind of marker affects the investigations on the mechanisms of epileptogenesis as well as the development of novel therapeutic approaches aimed to prevent or to mitigate the severity of the incoming epilepsy in humans. In this work, we provide evidence that in an experimental model of epileptogenesis that mimics the alteration of the blood-brain barrier permeability, a key-mechanism that contributes to the development of epilepsy in humans and in animals, the prolonged occurrence in the electrocorticograms (ECoG) of high rates of a nonlinear dynamical regimes known as intermittency univocally characterizes the population of experimental animals which develop epilepsy, hence it can be considered as the first biophysical marker of epileptogenesis.

## Introduction, Results and Discussion

According to the World Health Organization, 2.4 million people per year are diagnosed with epilepsy. Among these patients, about 40% develop epilepsy following the exposure to well-known risk factors as traumatic brain injury, stroke or infectious diseases, which can occur at any age. Following such insults, patients may develop the so-called post-traumatic epilepsy after a latent period known as epileptogenesis. Accordingly, many efforts are aimed to the identification of a marker of epileptogenesis, that is, a measurable event which is predictive of the emergence of this type of epilepsy. Indeed, from the perspective of the clinical application, two interdependent factors are strictly correlated with the identification of such a marker, i.e., i) the possibility to distinguish among all patients those who are actually at risk of developing the post-traumatic epilepsy and, for these at-risk patients, ii) the possibility to prevent the occurrence of the disease.

However, the possibility to distinguish patients which are actually at risk of developing epilepsy, is not necessarily the first medical priority, since there are no anti-epileptogenic therapies currently available. This point is crucial and leads to the further consideration that, in general, the availability of anti-epileptogenic therapies could reduce significantly the incidence of epilepsy in humans, since, in principle, a preventive therapy could be envisaged for any individual exposed to potential risk factors. Therefore, nowadays, the development of anti-epileptogenic therapies can be actually considered as the first clinically significant need, and the pre-clinical research plays a pivotal role in this context^1^ [and references therein].

Differently from the clinical perspective, it is important to consider that a marker of epileptogenesis useful in the pre-clinical research for the development of anti-epileptogenic therapeutics as well as to efficiently investigate on the mechanisms leading to the emergence of epilepsy, does not have to be necessarily predictive of the emergence of the disease at the level of each single experimental subject, although desirable. Indeed, when animal models are used to these aims, the common practice is to cluster the animals in experimental groups, each corresponding to a different treatment. Therefore, in this context, a necessary and sufficient condition for a measurable event in order to be defined as a marker of epileptogenesis is the capability to represent, at statistical level, only the experimental group with animals which develop epilepsy and, as such, to be sensitive to treatments that may show anti-epileptogenic properties.

Nowadays, despite the many efforts of the research in this field, there is no evidence of the existence of such a marker of epileptogenesis.

Pursuing this line of research, we recently characterized the nonlinear dynamics subtending the EEG during the early phases of epileptogenesis induced by status epilepticus in two different animal models^2^. We showed that a dynamical regime known as intermittency is prominent and persistent in the period following the epileptogenic insult. Intermittency is a dynamical state in which periods of apparent periodicity (known as laminar phases) alternate irregularly with periods showing dynamics driven by the emergence of one or more different, maybe chaotic, dynamic attractors^3^. This finding led us to hypothesize that the persistent occurrence of this nonlinear dynamical regime could be a model-independent marker of epileptogenesis. However, it was not possible to provide evidence of such assumption, due to the lack of proper experimental control groups, i.e., groups of animals that following an anti-epileptogenic treatment did not develop epilepsy, although they were exposed to the same epileptogenic insult.

In the present work, we tested the validity of such hypothesis by analyzing epidural electrocorticograms (ECoGs) related to a model of epileptogenesis induced by prolonged intracerebroventricular (ICV) infusion of serum albumin in mice. This experimental protocol simulates the extravasation of serum albumin in the cerebral parenchyma following the alteration of the permeability of the blood-brain barrier (BBB), a key-mechanism that contributes to the development of post-traumatic epilepsy in humans and in animals. This model offers several potentially translational advantages. Indeed, it is not based on the induction of epileptogenesis by eliciting the status epilepticus, an event that rarely occurs in human patients, although preserving the alteration of the BBB that occurs also as a consequence of the status epilepticus. Additionally, this model does not induce temporal sclerosis, cell loss and dispersion, similarly to what occurs in approximately 40% of patients affected by a common form of drug-resistant epilepsy as the temporal lobe epilepsy.

According to this model, the prolonged (7 days) ICV infusion of bovine serum albumin in mice induces epileptogenesis via the involvement of the TGF-β/ALK5 signalling, since the administration of an inhibitor of this specific signalling pathway (SJN2511) prevents the development of epilepsy^4^ [and references therein]. In the context of this experimental protocol, we analyzed the epidural ECoGs of i) mice (n = 7) which developed epilepsy following the treatment with 0.4 mM bovine serum albumin, ii) mice (n = 10) treated with artificial cerebrospinal fluid and 0.4 mM 70 KD dextran, representing the sham control group and iii) mice (n = 6) treated with bovine serum albumin and 300 μM SJN2511, which did not develop epilepsy.

For each mouse that developed epilepsy, we analyzed 12 seconds (4800 data points) of ECoG every 5 minutes of its respective period of epileptogenesis, up to 1 hour in advance of the onset of the first seizure. For each mouse that did not develop epilepsy, the same sampling procedure was extended up to 144 hours (6 days) from the beginning of ICV infusion of its respective treatment. All the selected epochs were analyzed by the recurrence quantification analysis (RQA), a powerful mathematical tool aimed to efficiently characterize the nonlinear dynamics embedded in short, noisy and nonstationary time-series, being the latter two features intrinsically associated with the nature of the EEG/ECoG signals^2^,^5^,^6^,^7^.

We measured the amount of laminar phases embedded in each selected epoch, thus measuring the amount of apparently periodic phases, which emerge following the occurrence of the intermittency regime. In the context of the RQA, not surprisingly, the main statistic correlated to the intermittency regime is called laminarity (LAM, for the formal definition of this variable see the ‘Methods’ section, paragraph ‘The Recurrence Quantification Analysis – General description’. See also the paragraph ‘Normalization of the variable LAM’ for the description of how this variable is expressed in the present work). We exploited the High-Throughput Computing technology (a.k.a. Grid Computing technology, see the ‘Methods’ section, paragraph ‘Computational resources and applications’) to considerably shorten the time required for the analysis of almost 32.000 epochs^8^.

**Figure 1:**
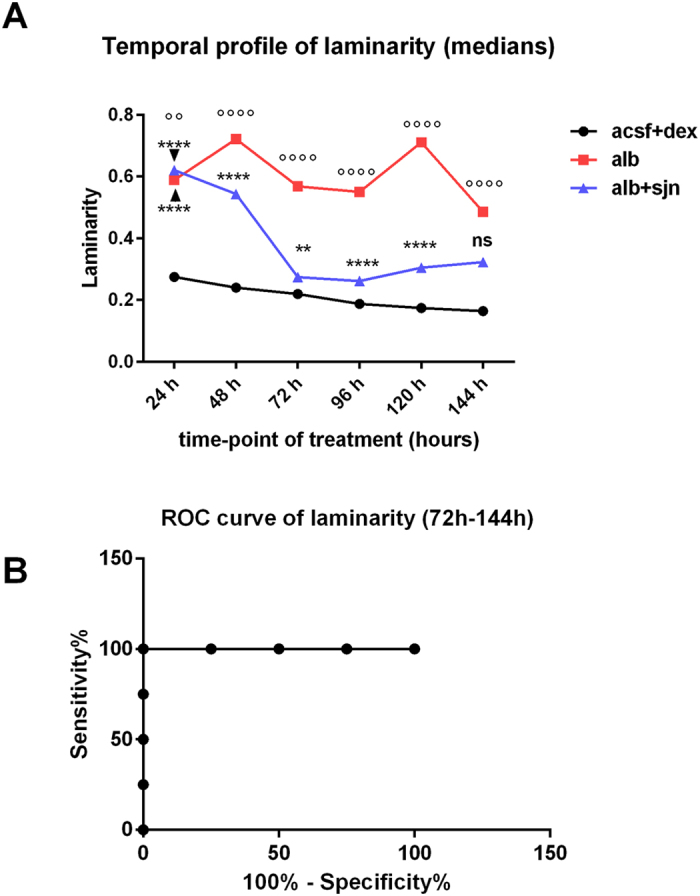
Graphical representation of results. (**A**) To depict the temporal profile of the variation of the amount of laminarity for each experimental group, we drew a line to connect data points representing the medians of values of laminarity calculated every 24 hours from the beginning of ICV infusion of vehicle (acsf + dex), albumin (alb) or albumin with SJN2511 (alb + sjn). Since none of the datasets passed the D’Agostino and Pearson omnibus normality test, statistical comparisons were made according to the nonparametric Kruskall-Wallis test, followed by Dunn’s multiple comparisons test. The detailed statistics are reported in the Supplementary Table S1. Legend of symbols in the (**A**): **p = 0.0036, ****p < 0.0001 vs. acsf + dex; °°p = 0.0010 vs. alb; °°°°p < 0.0001 vs. alb + sjn and vs. acsf + dex; ns: not significant vs. acsf + dex. (**B**) ROC analysis. The ROC curve was calculated considering the datasets starting from 72 hours after the beginning of infusion of albumin alone, or with SJN2511. Area under the ROC curve = 1, p = 0.0209.

The results of our analysis are graphically summarized in Fig. 1A. Full statistical details in the Supplementary Table S1. Following the epileptogenic insult, represented by the ICV infusion of albumin, the amount of LAM was high and persistent only in the experimental group of the animals that developed epilepsy. Conversely, the experimental group of animals that did not develop epilepsy, following the treatment with SJN2511, manifested a clear descending trend of the amount of LAM. This trend was such that already after 48–72 hours from the exposure to the same epileptogenic insult, the two experimental groups can be distinguished statistically. Accordingly, Fig. 1B, shows the ROC curve calculated considering the values of LAM measured starting from 72 hours after the beginning of ICV infusion of albumin, alone, or with SJN2511.

As recommended in the field of nonlinear time-series analysis, our results were challenged with a validation test (see the paragraph ‘Validation test of results’, in the ‘Methods’ section), which confirmed that our measurements are the expression of actual nonlinear dynamics. Accordingly, in the Supplementary Tables S2 and S3, we provide the detailed statistics of the other two variables strictly correlated to the variable LAM in the context of the RQA, i.e., VMAX and TT (for the formal definition of these variables see the ‘Methods’ section, paragraph ‘The Recurrence Quantification Analysis – General description’). Both variables change coherently with the trend of the variable LAM, thus confirming our finding. Therefore, our results provide evidence that, following an insult that mimics a dysfunction of the BBB, a sustained and prolonged nonlinear dynamical regime of laminar/intermittency type is the biophysical hallmark of epileptogenesis.

It is of interest to notice that this nonlinear regime is pervasive in the context of the brain dynamics involved in the epileptic phenomena. Indeed, it was shown emerging in the EEGs during ictal activities in epileptic patients as well as in rodents during the progression of the SE^9^,^10^. However, this regime is not only associated to ictal events but characterizes the brain electrical activity which emerges after the exposure to risk factors as the SE^2^ and the extravasation of serum albumin in the brain parenchyma (this study). In these cases, a marked laminar/intermittency regime occurs without any apparent seizure activity and characterizes the period of the epileptogenesis. We stress that the qualitative similarity of these findings occurs despite the remarkable differences among the types of epileptogenic insults, the presence or less of neurodegenerative phenomena and the different brain areas involved in the EEG recordings^2^,^4^. Therefore, the present work not only introduces the first nonlinear biophysical marker of epileptogenesis, but strengthens the hypothesis that this could be a model-independent marker, hence a potential general marker of epileptogenesis that could be detected also in the EEG/ECoG of the patients at risk of developing the post-traumatic epilepsy.

It is difficult to interpret the functional meaning of the emergence of such dynamic in the light of current evidence, without further investigations. However, it is of interest to notice that ictogenic brain areas are characterized by a high rate of occurrence of spatially distributed ‘micro-seizures’^11^. Since the intermittency may characterize the dynamics of ictal events^10^, it is conceivable that the emergence of the intermittency regimes during the period of the epileptogenesis could be the expression of such ‘micro-seizures’, which originate from an epileptogenic network under formation^11^,^12^.

We are confident that our findings can significantly contribute to the discovery of the fundamental mechanisms leading to the emergence of epilepsy, and to improve and speed up the development of anti-epileptogenic therapeutic interventions to benefit patients at risk of post-traumatic epilepsy. Additionally, these findings could open the way to the identification of patients at risk of developing post-traumatic epilepsy, by considering the amount of laminarity/intermittency of their EEG/ECoG signals.

## Methods

### Model of epileptogenesis

We analyzed the epidural electrocorticograms (ECoGs) recorded in an experimental model during an investigation on the mechanisms of epileptogenesis induced by the alteration of the blood-brain barrier (BBB). This model is fully described in the reference paper^4^. All animal procedures were performed in accordance with the guidelines and the regulations approved by the animal care and use ethical committees at the Ben-Gurion University of the Negev, Beer-Sheva.

Briefly, two-month-old mice (FVB-N) were surgically implanted with osmotic micro-pumps, in order to deliver the treatments of interest in the right lateral cerebral ventricle, and with two screws inserted in holes drilled through the skull over the cortex (0.5 mm anterior, 2.5 later to lambda). The screws acted as electrodes, thus generating the epidural ECoGs.

According to this model, 7 days of intracerebroventricular (ICV) infusion of a physiological serum concentration of albumin (bovine serum albumin, 0.4 mM) induces epileptogenesis via the TGF-β/ALK5 signalling, since the administration of SJN2511, a specific TGF-β/ALK5 signalling inhibitor, prevents the development of epilepsy^4^ [and references therein].

### Selection criteria of the period of epileptogenesis

On average, epileptogenesis for mice infused with albumin lasted 5.14 ± 1.70 (mean ± SD) days. More specifically, during the 7 days of infusion of albumin, 5 animals out of 7 developed epilepsy. For these mice, we considered the ECoGs up to 1 hour in advance of the onset of the first seizure. For the two animals that developed epilepsy after withdrawal of album infusion, we considered the ECoGs up to 144 hours (6 days) of albumin infusion. This prevented possible effects due to the albumin withdrawal, and preserved a numerosity of at least 4 mice (57% of the initial group) for this experimental group for the time-point of albumin infusion corresponding to 144 hours, for statistical comparisons.

### Selection criteria of epochs for nonlinear analysis and pre-processing of ECoG signals

We considered the epidural ECoGs of i) mice (n = 7) developing epilepsy following the treatment with 0.4 mM bovine serum albumin, ii) mice (n = 10) treated with artificial cerebrospinal fluid with 70 KD dextran, representing the sham control group and iii) mice (n = 6) treated with bovine serum albumin and 300 μM SJN2511, which did not develop epilepsy.

The ECoGs, originally sampled at 1000 Hz, were re-sampled at 400 Hz. In order not to alter/filter-out any potential nonlinear structure embedded in the original signals, neither filtering nor pre-processing of any kind were applied to ECoGs^13^. For each mouse that developed epilepsy, we analyzed 12 seconds (4800 data points) of ECoG every 5 minutes of the period of epileptogenesis, as defined in the previous paragraph. For each mouse that did not develop epilepsy, the selection of the 12-second epochs of ECoG every 5 minutes was extended up to 144 hours (6 days) from the beginning of ICV infusion of its respective treatment.

All the selected epochs were analyzed by the recurrence quantification analysis, since this mathematical tool is particularly suitable to detect and to measure nonlinear dynamics embedded in short, noisy and nonstationary time-series^2^,^5^,^6^,^7^.

### The Recurrence Quantification Analysis

#### General description

A fundamental property of nonlinear dynamical systems is the recurrence of states, which become arbitrarily close to each other after a sufficient time^14^. This property inspired Eckmann and colleagues^15^ to introduce the technique of the Recurrence Plot (RP), which allows representing the time evolution of the states of a nonlinear dynamical system with a two-dimensional representation of its recurrences. Indeed, a RP is the graphical representation of a squared matrix (the recurrence matrix), where the recurrence of a state at two different times is marked according to a binary decision, e.g., one and zero, to denote if this state is recurrent (one) or not (zero).

Graphically, a RP exhibits characteristic large-scale and small-scale patterns, denoted as typology and texture, respectively^15^. The small-scale structures of texture consist of single dots, diagonal lines and vertical and horizontal lines. Single, isolated recurrence points can occur if states are rare, and can be due to chance or noise. A diagonal line occurs when a sequence of states (a.k.a. trajectory in the phase space of the system) visits the same region of the phase space at different times and its length represents the period during which two trajectories are sufficiently close each other and show a similar time evolution. A vertical (horizontal) line marks a time length in which a state does not change or changes very slowly, as in laminar states (intermittency). Therefore, for a given nonlinear dynamical system, the associated RP allows to infer visually some important dynamical properties of the system, even in case of short, noisy and nonstationary datasets.

A fundamental step forward for the application of the RPs, not only as a graphical tool, but also as a quantitative analytical tool, was the development of the Recurrence Quantification Analysis (RQA)^5^. The RQA is a powerful analytical technique based on the mathematical definition of variables introduced to measure some important properties of a nonlinear dynamical system, quantifying the small-scale structures of the corresponding RP.

At the present state of development of the RQA, several variables have been introduced^3^,^5^ (see also http://www.recurrence-plot.tk). In this study, we considered the variables that have been defined as follows:

- Recurrence Rate (REC), which represents the density of recurrence points in a RP. The recurrence rate corresponds with the probability that a specific state will recur and is expressed as


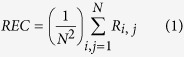


with *R*_*i, j*_ = Θ (RAD_i_ − ||x_i_ − x_j_||), x_i_ ∈ ℜ^m^, i, j = 1…*N*, where *N* is the number of considered states, x_i_, RAD_i_ is a threshold distance (a.k.a. *radius*), || · || a norm and Θ(·) the Heaviside function;

- Laminarity (LAM), which represents the fraction of recurrence points forming vertical lines. Vertical lines represent unchanged or slowly changing states of the system and are associated to laminar states. LAM is defined as


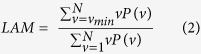


where *v* is the vertical line length considered when its value is ≥*v*_*min*_ and *P* (*v*) is the probability distribution of vertical line lengths;

– Longest Vertical Line (VMAX), which represents the length of the longest vertical line


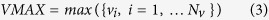


– Trapping time (TT), which represents the average length of the vertical lines


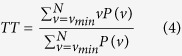


The computation of RQA variables requires the preliminary reconstruction of the trajectories of the system under investigation in a phase space, according to the time-delay embedding procedure, introduced by Takens^16^. According to the Takens’ theorem, the topological features of any high-dimensional system, consisting of multiple coupled variables, can be reconstructed by measuring just a single variable of that system^13^,^16^. In other words, a single observable of the system is sufficient to allow the reconstruction of the trajectories in a high-dimensional phase space that preserves the dynamical properties of the system (topological invariants), although this reconstructed high-dimensional phase space is qualitatively different from the ‘true’ phase space. A major advantage of the Taken’s theorem is that the ‘true’ phase space can be totally unknown, as it is often the case in the context of biological systems.

A single point of a trajectory in a phase space reconstructed according to the Takens’ theorem represents a vector made of *m* time points selected from the time series, where two consecutive time points are delayed by a predetermined time lag (a.k.a. *time delay*). The number (*m*) of time points chosen as components of vectors is referred to as the *embedding dimension* of the time-series. Therefore, the reconstructed phase space has *m*-axis, one for each of the *m* components of the vector. Besides the embedding dimension and the time delay, the RQA requires to set the radius (RAD), which represents a small *m*-dimensional volume of the reconstructed phase space that acts as a threshold by which all points included in this volume are defined as recurrent points and, accordingly, marked as such in the recurrence matrix.

#### Preliminary scaling analysis and determination of parameters for the RQA

As described in the previous paragraph, for an accurate RQA a crucial step is the determination of the embedding dimension, the RAD and, to a lesser extent, the time delay. To this aim, we accomplished a preliminary scaling analysis of subsets of epochs (n = 46, two epochs randomly selected from each mouse), according to the strategy suggested by Webber^13^. The scaling analysis allows to determine sets of values of embedding dimension and RAD for which the nonlinear deterministic patterns of the time series under investigation are sufficiently unveiled. A significant deterministic pattern to consider as a reference for an appropriate choice of parameters is the exponential scaling behaviour of the variable REC vs. the variable RAD. This scaling behaviour is graphically manifested as a linear tract on a log-log plot of REC vs. RAD and is usually expected to occur for low percentage values of REC, typically from 0.05% to 2%.

According to our previous experience^2^, we set to 12 the value of the embedding dimension, and the REC was set to the sequence of values 0.1, 0.5, 1.0, 2.0, 5.0 and 10, expressed as % of the max distance of the recurrence matrix.

**Figure 2:**
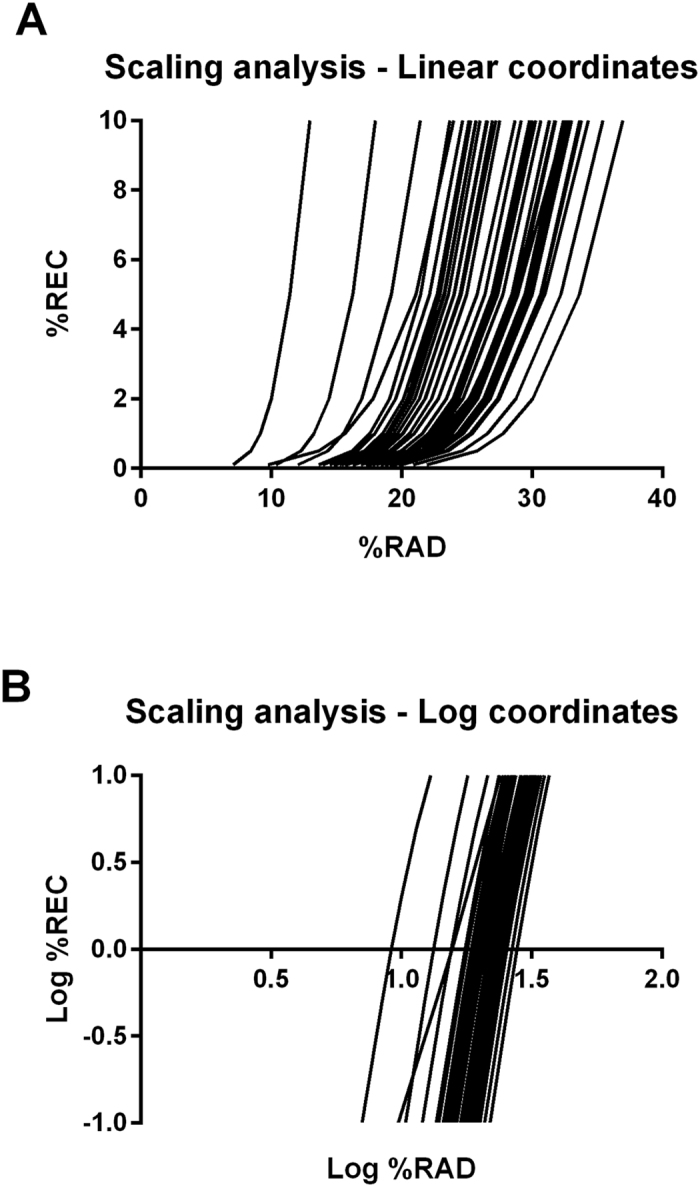
Preliminary scaling analysis. A fundamental prerequisite for an appropriate estimation of the RQA variables is a sparse percentage of REC, usually within the range 0.05–2%. In logarithmic coordinates, this range of values is expected to be within the linear tract of the exponential scaling function of the variable %REC vs. the variable %RAD. When this prerequisite is satisfied, the estimate of the RQA variables can be made with any constant value of the %RAD that yields such a sparse % REC. However, this procedure is not appropriate in our experimental conditions. Indeed, the exponential relationship depends on the epoch considered, thus giving rise to a broad spectrum of functions (**A**). Consequently, a constant value of the %RAD may be appropriate for some epochs but totally inadequate for all the other ones (**B**). Our preliminary scaling analysis visually highlights a linear tract (in logarithmic coordinates), common to all the epochs considered, for values of %REC around 1.0 (0.0 in the log-log plot, **B**). Therefore, we set the %REC to the constant value 1 (corresponding to a sparse recurrence matrix), and considered the %RAD as a variable.

As we noticed also in our previous investigation, the common approach of choosing a fixed RAD and then to determine the RQA variables may not be the most appropriate method for the analysis of EEG/ECoG epochs. Indeed, the graphs of %REC vs. %RAD (Fig. 2A,B) clearly show that the exponential relationship between these two variables fluctuates amply, from epoch to epoch. In particular, the variability of the RAD is such that a fixed RAD may not necessarily belong to the domain of the function REC vs. RAD and, even in this case, it does not necessarily imply that the percentage of REC is low and within the linear range of the log-log plot of %REC vs. %RAD, a stringent requirement for an appropriate estimation of the RQA variables. Therefore, also in this study, we considered the RAD as a variable and the REC as a parameter that we set to 1%, a percentage that assured a sparse recurrence matrix and the preservation of the linear relationship of log-log plot of REC vs. RAD in all epochs considered (Fig.2B).

We decided not to set a time delay common to all epochs and to determine the appropriate time delay for each epoch by considering the first local minimum of the mutual information function. The value of the time delay was used also for the determination of the Theiler’s window^17^, that we calculated according to Gao^18^, i.e., [(*m−1*) * (*time delay*)], thus reducing the influence of tangential motion in the estimate of the RQA variables^3^.

The recurrence matrix was normalized to the maximum distance^13^ and the variable RAD was expressed as percentage of the recurrence matrix, whereas the distance between vectors was computed as Euclidean distance. Additionally, the minimum diagonal line length was set at a conservative value of 5, since the observational noise was shown to significantly affect the amount of diagonal lines with spurious diagonal segments with length ≤4^19^. The minimum vertical line length was set at the default value of 2, since there are no indications available on the effect of noise on this parameter.

**Figure 3:**
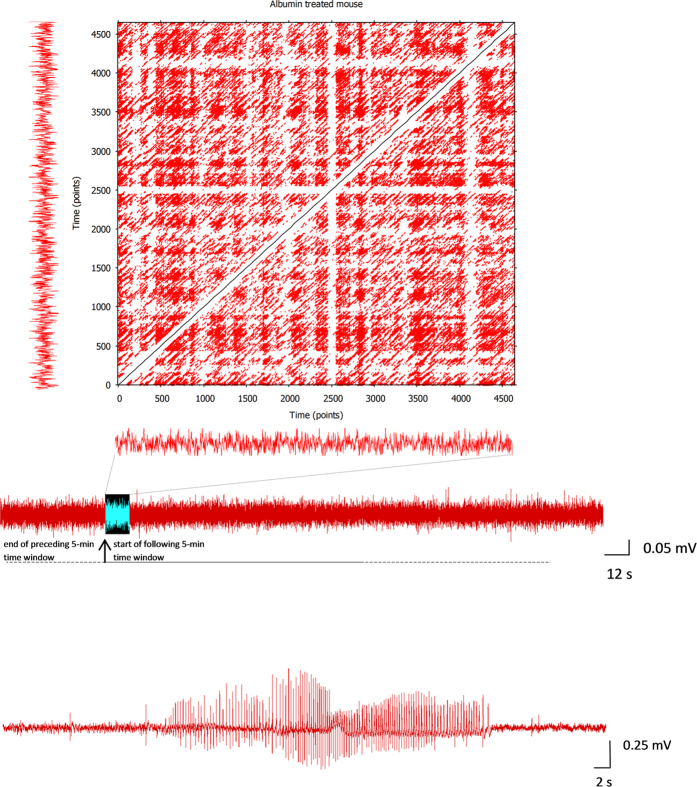
Recurrence Plot of an ECoG epoch selected according to our sampling protocol. The Recurrence Plot (RP) shown in the figure is the graphical representation of the recurrence matrix associated to an ECoG epoch selected according to our sampling protocol, i.e., 12 seconds (4800 data points) every 5 minutes of ECoG (middle trace). In this example, the ECoG was recorded during the period of epileptogenesis of an animal that developed epilepsy following the exposure to ICV infusion of albumin. In the figure, the selected epoch for the construction of the RP is represented by the highlighted portion of the ECoG. In the RP, the occurrence of laminarity states generates dark areas, with different extension, which are made of adjacent points positioned vertically and/or horizontally, sometimes loosely similar to squares and rectangles. The RQA variable LAM is aimed at measuring the percentage of the recurrent points which form vertical lines in the RP. Another important graphical aspect of RPs is the presence of diagonal lines which are associated to the degree of determinism of the time-series. In this case, the RQA variable DET is aimed at measuring the percentage of the recurrent points which form diagonal lines in the RP (see also the Supplementary Discussion). The RPs are graphically symmetric respect to the diagonal representing the line of identity (LOI) of the squared recurrence matrix. The width of the white stripe along the LOI depicts the extension of the Theiler’s window, which usually varies from epoch to epoch. In the context of our analytical protocol it is important to consider that the RP associated to each ECoG epoch does not correlate with the actual amount of laminarity (and determinism) of the time-series, since the range of variation of the RAD is limited by the constrain to vary until the imposed fixed amount of recurrences is reached (1%). See the ‘Methods’ section, paragraph ‘Normalization of the variable LAM’, for details. The lower ECoG trace represents a typical ictal event (seizure) occurring in the animals that developed epilepsy.

**Figure 4:**
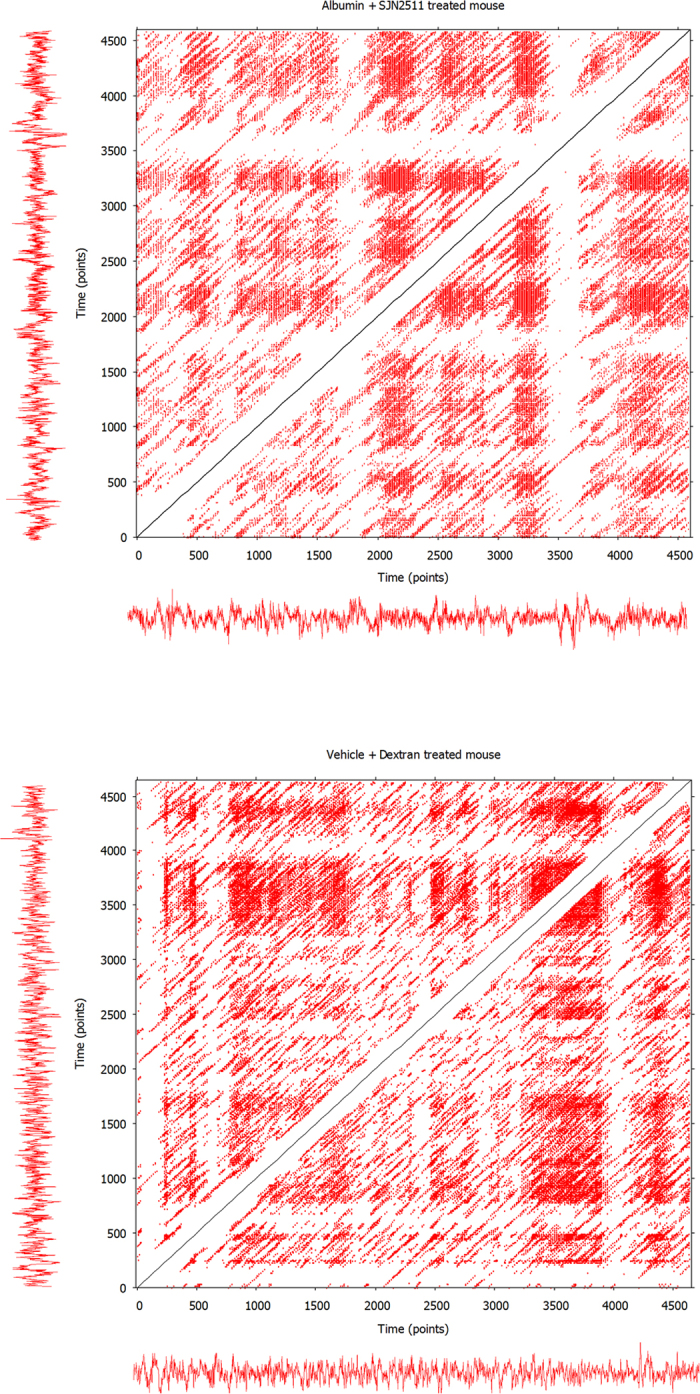
Further examples of Recurrence Plots. The upper RP is associated to an ECoG epoch of a mouse treated with albumin + SJN2511, selected after approximately 32 hours from the beginning of the treatment. The lower RP is associated to an ECoG epoch of a mouse treated with vehicle + Dextran, selected after approximately 16 hours from the beginning of the treatment. Very different patterns of recurrent points can emerge in a RP, thus giving rise to different amounts of laminarity (vertical lines) and determinism (diagonal lines, see also the Supplementary Discussion), which usually change from epoch to epoch. Nevertheless, we stress again that the RPs are not correlated with the actual amount of laminarity and determinism of the time-series. This is due to our analytical approach, as explained in the ‘Methods’ section, paragraph ‘Normalization of the variable LAM’. See also the legend of Fig. 1. Therefore, we discourage the reader to compare the RPs to each other in order to have a visual representation of how the amount of occurrence of laminarity states varies among the experimental groups.

In order to clarify the meaning of the RQA variables in the context of a RP, in Figs 3 and 4 we provide examples of RPs that are associated to some ECoG epochs selected from the experimental groups.

#### Normalization of the variable LAM

It is necessary to consider that when the RAD is a variable and the REC is a fixed parameter, the measurements of the variable LAM, *per se*, do not correlate with the actual amount of laminarity of the time-series. Indeed, the range of variation of the RAD is limited by the constrain to vary until the imposed fixed amount of recurrences is reached, that is 1% in our case. This leads to biases of the estimate of the amount of LAM of the time-series. However, this drawback can be easily overcome by normalizing the value of the LAM to the value of the RAD, that is, by expressing the amount of laminarity as the ratio LAM/RAD^2^.

#### Validation test of results

In principle, the values of the RQA variables may be also the expression of a Gaussian linear stochastic process, hence the necessity to ascertain whether they can be reasonably attributed to ongoing nonlinear processes that actually subtend the behaviour of the system under investigation. The surrogate data technique, as introduced by Theiler^17^,^20^, is a common practice to this aim. It is based on the generation of (at least 19) surrogates data for each time-series for which the RQA variables were calculated, by the technique of the inverse Fourier transform with phase randomization. This technique creates random epochs that, approximately, preserve the power spectrum as well as the same distribution of values of the original epoch, but devoid of any nonlinear determinism. However, this technique of generation of surrogate data cannot be indiscriminately applied to all signals. Indeed, an implicit assumption of this technique is that the time-series, from which the surrogates are constructed, be devoid of strong periodic components. When this assumption is not satisfied, two major drawbacks occur. Indeed, since the power spectrum of each surrogate does not match with that of the original epoch exactly, spurious detection of nonlinearities may occur. Conversely, since a phase-randomized surrogate data of a periodic signal, is itself a periodic signal, the periodicities arising from nonlinear limit cycles, may not be detected.

**Figure 5:**
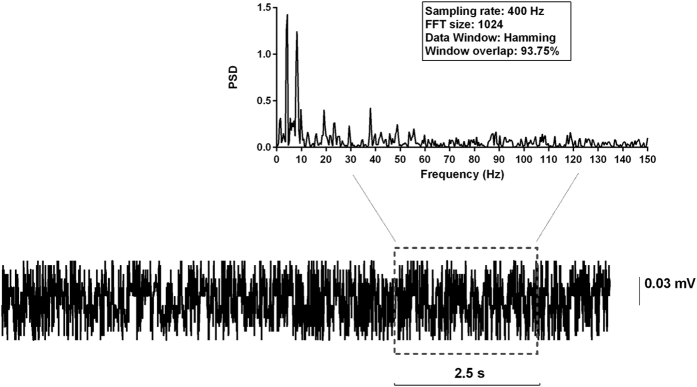
Detection of periodic components in ECoGs. Power spectrum density (PSD) of an epoch randomly selected among those of the experimental group of animals which did not develop epilepsy, following 96 hours of perfusion with SJN2511. Peaks of periodic components are clearly evident.

We consistently detected periodic components in randomly selected epochs (Fig. 5), hence the application of the surrogate data technique, as described above, is not recommended as validation test in our conditions.

**Figure 6:**
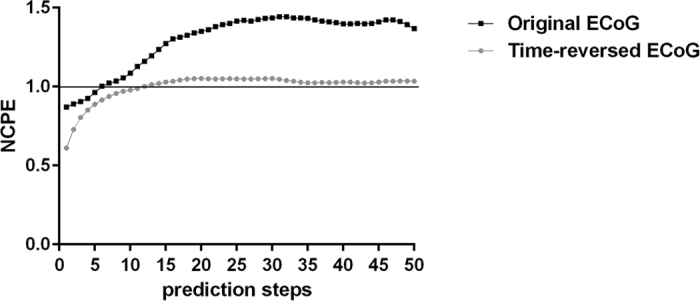
Evidence of nonlinearity in an ECoG epoch. Example of nonlinear cross-prediction errors (NCPEs), calculated for 50 prediction steps, for an ECoG epoch with underlying nonlinear dynamics. All the NCPEs are expressed as normalized values respect to the standard deviation of the dataset to be predicted.

Nevertheless, evidence of nonlinearity in a time series with significant periodic components, can be gained considering two properties of nonlinear dynamical systems, i.e., the deterministic nature and the time-irreversibility^21^,^21^. Since a nonlinear dynamical system is deterministic, a certain degree of predictability is intrinsic to the system itself. Therefore, a subset of data of the system can be modelled in order to predict another subset of data of the same system. The goodness of such prediction can be estimated by measuring a prediction error (nonlinear cross-prediction error, NCPE), which increases the more the prediction is projected into the near future. The predictability of nonlinear dynamical systems is asymmetric respect to time, in that their deterministic nature allows short-term predictions that do not hold true for the corresponding time-reversed dataset. In this case, the NCPE converges to the standard deviation of the time-series (Fig. 6).

Keeping in consideration the two properties mentioned above, we exploited the algorithm *xzero*^23^ for the calculation of the NCPEs for each epoch analyzed in our investigation. Specifically, we calculated the NCPEs when i) the epoch is split in two datasets (two halves), and the first one is used to predict the second one by approximating the dynamics locally by a constant, that is, by fitting a zeroth order model, and ii) the whole epoch is used to predict the same time-reversed epoch. The values of the NCPEs were calculated for up to 50 prediction steps into the future and the embedding dimension was set to 12, as it was used during the RQA analysis. All the NCPEs are expressed as normalized values respect to the standard deviation of the dataset to be predicted.

To gain evidence of nonlinearity with an automated procedure applied to the nearly 32000 epochs selected for our investigation, for each epoch we considered the two maximum values of the NCPE, i.e., the first one, related to the prediction of the second-half dataset (NCPE-det) and, the second one, related to the prediction of the time-reversed epoch (NCPE-trev). Then, for each experimental group, at each time-point of epileptogenesis, we tested the statistical significance of the difference between the NCPEs-det dataset vs. the NCPEs-trev dataset, by the nonparametric Mann-Whitney test, since none of the datasets was normally distributed, according to the D’Agostino and Pearson omnibus normality test (P < 0.0001 for all datasets).

**Figure 7:**
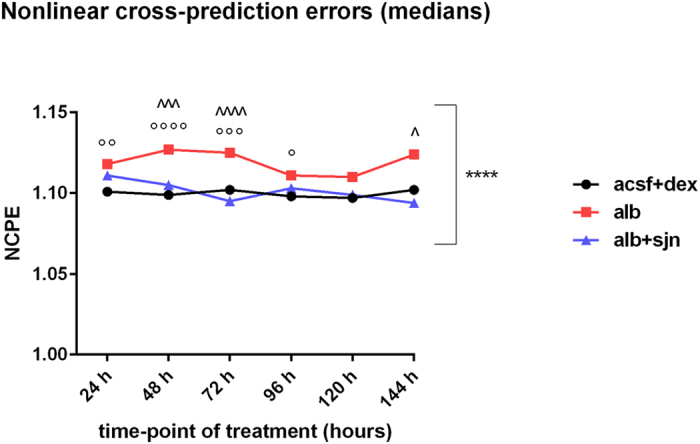
Results of the validation test. Temporal profile of the medians of the NCPEs-det. For each experimental group, at each time-point of treatment, indications of ongoing nonlinear processes were detected, occurring at different extent. In particular, following a potentially epileptogenic insult, as the alteration of the BBB permeability induced by the ICV infusion of albumin, the emergence of nonlinear phenomena is more pronounced in the experimental group of animals which will develop epilepsy (alb). Legend of symbols: ****p < 0.0001 for each experimental group, at each time-point, vs. the respective dataset of NCPEs-trev, by Mann-Whitney test; °p < 0.05, °°p < 0.01, °°°p < 0.001, °°°°p < 0.0001 vs. acsf + dex; ^p < 0.05, ^^^p < 0.001, ^^^^p < 0.0001 vs. alb + sjn, by Kruskall-Wallis test, followed by Dunn’s multiple comparisons test in case of significant variation of the medians (p < 0.05).

The results are summarized in Fig. 7, which shows the temporal profile of the medians of the NCPEs-det, for all experimental groups. As compared to their NCPEs-trev, all the NCPEs-det were statistically different (p < 0.0001, for all datasets, by Mann-Whitney test, Fig. 7 - square bracket), with medians significantly higher than 1, i.e., with a short-term prediction error that is significantly greater than the standard deviation of the predicted dataset, a property of the nonlinear dynamical systems. It is important to notice that results based on the NCPE technique, must be correctly interpreted^21^. Indeed, these results provide evidence of time asymmetry in ECoG epochs, thus evidence of nonlinearity. However, this finding does not represent a measure of the amount of nonlinearity in the underlying system. Rather, it provides an indication of the strength of nonlinearity in the system, so that the stronger the time asymmetry, the stronger the indication of nonlinearity.

As shown in Fig. 7, additional statistics (Kruskall-Wallis test, followed by the Dunn’s multiple comparisons test) highlighted that evidence of nonlinearity is stronger in the experimental group of the animals that develop epilepsy. It is of interest to notice that during the early phases of epileptogenesis (24–72 hours), the NCPEs-det can statistically discriminate between the experimental group of animals treated with albumin and that of animals treated with albumin and SJN2511, similarly to what occurs with the RQA variables (see the main text and Supplementary Discussion) during the same period. This observation may suggest that also the NCPE technique can be useful, by itself, as an analytical tool to discriminate among experimental groups, a finding that is worth investigating also in other experimental contexts.

The general conclusion, derived from the application of the NCPE technique as validation test of our results, is that the experimental groups have ongoing nonlinear phenomena, which occur at different extent, depending on the experimental group and the period of epileptogenesis. Therefore, the values of the RQA variables can be reasonably considered as the expression of actual nonlinear processes.

#### Computational resources and applications

The usage of High-Throughput-Computing Technology, a.k.a. Grid Computing Technology, has considerably shortened the time for calculation of the RQA variables. A Grid Computing infrastructure represents a network of geographically distributed computational resources that allow the execution of applications on distributed computational and storage resources across the Internet. The computational resources used in this study were those of the INFN (National Institute of Nuclear Physics), which are part of the Italian Grid Infrastructure (IGI) that is fully integrated in the European Grid Infrastructure (EGI), the Europe’s leading grid computing infrastructure co-funded by the European Union in the context of the 6th and 7th Framework Program. The middleware, i.e., the software specifically developed to manage the workload across the Grid Computing infrastructure, was gLite v. 3.1–3.2 and the interactive sessions were made by a Scientific-Linux command-line interface.

The RQA was accomplished by the applications RQS and RQH, as developed by Webber^24^ (freely available for Windows operating systems at http://homepages.luc.edu/~cwebber). However, in order to implement the Theiler’s window and to make the applications executable in the Grid Computing environment, the applications were adapted, with minor modifications, as ANSI C code. Other applications exploited in this work were *mutual, minima, extrema* and *xzero*, all included in the TISEAN software package^23^ (freely available at http://www.mpipks-dresden.mpg.de/~tisean).

#### Statistics and graphical representations

Statistical tests were performed by the software package GraphPad Prism 6 (GraphPad Software Inc., San Diego, California, USA). None of the datasets was normally distributed, according to the D’Agostino and Pearson omnibus normality test (P < 0.0001 for all datasets). Therefore, we used the nonparametric Kruskal-Wallis test, followed by the Dunn’s multiple comparisons test, in case of significant variation of medians (P < 0.05), or the Mann-Whitney test, as appropriate. For illustrative purposes, the datasets were represented with medians, jointed by lines drawn to guide the eye, in order to depict the temporal profile of the variations of the variables of interest. The graphs also report the symbols of statistical significance, according to the results of the statistical tests.

## Additional Information

**How to cite this article**: Rizzi, M. *et al*. Following a potential epileptogenic insult, prolonged high rates of nonlinear dynamical regimes of intermittency type is the hallmark of epileptogenesis. *Sci. Rep.* **6**, 31129; doi: 10.1038/srep31129 (2016).

## Supplementary Material

Supplementary Information
