# Corrigendum: Following a potential epileptogenic insult, prolonged high rates of nonlinear dynamical regimes of intermittency type is the hallmark of epileptogenesis

**DOI:** 10.1038/srep35510

**Published:** 2016-10-14

**Authors:** Massimo Rizzi, Itai Weissberg, Dan Z. Milikovsky, Alon Friedman

Scientific Reports 6: Article number: 3112910.1038/srep31129; published online: 08 04 2016; updated: 10 14 2016

The Acknowledgements section in this Article is incorrect.

“The research leading to these results has received funding from the European Union’s Seventh Framework Program (FP7/2007–2013) under grant agreement no. 602102 (EPITARGET). We wish to thank Prof. Charles Webber, who generously provided the source codes of RQA applications used in this work, and Dr. Francesca Falcetta for the valuable help provided for the data format conversion. We also wish to thank Dr. Giuseppe La Rocca (Italian National Institute of Nuclear Physics, Division of Catania, Italy) and Prof. Giuseppe Barbera (Italian National Institute of Nuclear Physics, Division of Catania and Department of Physics and Astronomy of the University of Catania, Italy) for their technical assistance on the usage of Grid Computing resources and services provided by the Italian Grid Infrastructure (IGI, http://www.italiangrid.it/). We are grateful to ARCEM - Associazione Italiana per la Ricerca sulle Patologie Cerebrali e del Midollo Spinale that supported the whole cost of publication of this work”.

Should read:

“The research leading to these results has received funding from the European Union’s Seventh Framework Program (FP7/2007–2013) under grant agreement no. 602102 (EPITARGET). We wish to thank Prof. Charles Webber, who generously provided the source codes of RQA applications used in this work, and Dr. Francesca Falcetta for the valuable help provided for the data format conversion. We also wish to thank Dr. Giuseppe La Rocca (Italian National Institute of Nuclear Physics, Division of Catania, Italy) and Prof. Roberto Barbera (Italian National Institute of Nuclear Physics, Division of Catania and Department of Physics and Astronomy of the University of Catania, Italy) for their technical assistance on the usage of Grid Computing resources and services provided by the Italian Grid Infrastructure (IGI, http://www.italiangrid.it/). We are grateful to ARCEM - Associazione Italiana per la Ricerca sulle Patologie Cerebrali e del Midollo Spinale that supported the whole cost of publication of this work”.

