# Supplementary material for: Following a potential epileptogenic insult, prolonged high rates of nonlinear dynamical regimes of intermittency type is the hallmark of epileptogenesis
Source: Sci Rep. 2016 Aug 4;6:31129. doi: 10.1038/srep31129 (PMC4973227; doi:10.1038/srep31129)
Supplement: Supplementary Information [file srep31129-s1.pdf]

Supplementary Information

Following a potential epileptogenic insult, prolonged high rates of nonlinear dynamical regimes of intermittency type is the hallmark of epileptogenesis

Massimo Rizzi[1,*], Itai Weissberg[2], Dan Z. Milikovsky[2], Alon Friedman[2,3]

[1]Department of Neuroscience, IRCCS - Istituto di Ricerche Farmacologiche Mario Negri, Via G. La Masa 19, 20156 Milan, Italy

[2]Departments of Physiology and Cell Biology, Cognitive and Brain Sciences, Zlotowski Center for Neuroscience, Ben-Gurion University of the Negev, Beer-Sheva 84105, Israel

[3]Department of Medical Neuroscience, Faculty of Medicine, Dalhousie University, Halifax, Nova Scotia B3H 4R2, Canada

*Corresponding author: M. Rizzi, Dept. Neuroscience, IRCCS - Istituto di Ricerche Farmacologiche Mario Negri, Via G. La Masa 19, 20156 Milan, Italy

e-mail address: massimo.rizzi@marionegri.it

Supplementary Table S1. Detailed statistics of the variable LAM

	Time-point of treatment (hours)					
	**24 h**	**48 h**	**72 h**	**96 h**	**120 h**	**144 h**
**Number of values**						
acsf+dex	2554	2502	2000	2590	2602	2658
alb	1853	1794	1327	1542	1261	663
alb+sjn	1627	1638	1254	1530	1270	1033
**Median, percentile**						
acsf+dex						
25% Percentile	0.0897	0.07513	0.07322	0.0519	0.04932	0.0471
Median	0.2751	0.2401	0.2195	0.1876	0.1741	0.1641
75% Percentile	0.6111	0.5593	0.5489	0.5478	0.4747	0.5234
alb						
25% Percentile	0.2942	0.3089	0.2596	0.2577	0.3087	0.1351
Median	0.5888	0.7215	0.5686	0.5502	0.711	0.4859
75% Percentile	0.9417	1.119	1.046	1.075	1.126	0.9734
alb+sjn						
25% Percentile	0.07659	0.07281	0.08835	0.108	0.1357	0.002458
Median	0.621	0.5434	0.2741	0.2615	0.3048	0.3233
75% Percentile	1.201	0.951	0.669	0.5813	0.6188	0.7285
**Kruskal-Wallis test**						
P value	< 0.0001 ****	< 0.0001 ****	< 0.0001 ****	< 0.0001 ****	< 0.0001 ****	< 0.0001 ****
Kruskal-Wallis statistic	385.7	650.2	428.8	625.7	715.1	189.2
**Dunn's multiple comparisons test**						
acsf+dex Vs. alb - Adjusted P Value	< 0.0001 ****	< 0.0001 ****	< 0.0001 ****	< 0.0001 ****	< 0.0001 ****	< 0.0001 ****
acsf+dex Vs. alb+sjn - Adjusted P Value	< 0.0001 ****	< 0.0001 ****	0.0036 **	< 0.0001 ****	< 0.0001 ****	0.1216 ns
alb Vs. alb+sjn - Adjusted P Value	0.001 **	< 0.0001 ****	< 0.0001 ****	< 0.0001 ****	< 0.0001 ****	< 0.0001 ****

Legend: ns, not significant

Supplementary Table S2. Detailed statistics of the variable VMAX

	Time-point of treatment (hours)					
	**24 h**	**48 h**	**72 h**	**96 h**	**120 h**	**144 h**
**Number of values**						
acsf+dex	2576	2522	2005	2594	2623	2678
alb	1871	1806	1337	1571	1300	674
alb+sjn	1646	1653	1266	1552	1277	974
**Median, percentile**						
acsf+dex						
25% Percentile	12	11	11	11	11	11
Median	16	16	15	15	15	15
75% Percentile	22	21	21	20	20	20
alb						
25% Percentile	15	15	15	16	16	14
Median	19	21	19	20	21	18
75% Percentile	24	27	26	25	25	24
alb+sjn						
25% Percentile	13	13	12	12	12	9
Median	19	18	16	16	17	15
75% Percentile	28	25	23	22	22	21
**Kruskal-Wallis test**						
P value	< 0.0001 ****	< 0.0001 ****	< 0.0001 ****	< 0.0001 ****	< 0.0001 ****	< 0.0001 ****
Kruskal-Wallis statistic	208.2	260.1	172.4	412.0	401.8	121.6
**Dunn's multiple comparisons test**						
acsf+dex Vs. alb - Adjusted P Value	< 0.0001 ****	< 0.0001 ****	< 0.0001 ****	< 0.0001 ****	< 0.0001 ****	< 0.0001 ****
acsf+dex Vs. alb+sjn - Adjusted P Value	< 0.0001 ****	< 0.0001 ****	< 0.0001 ****	< 0.0001 ****	< 0.0001 ****	0.4671 ns
alb Vs. alb+sjn - Adjusted P Value	0.173 ns	< 0.0001 ****	< 0.0001 ****	< 0.0001 ****	< 0.0001 ****	< 0.0001 ****

Legend: ns, not significant

Graphical representation of statistics of the variable VMAX, as reported in Supplementary Table S2

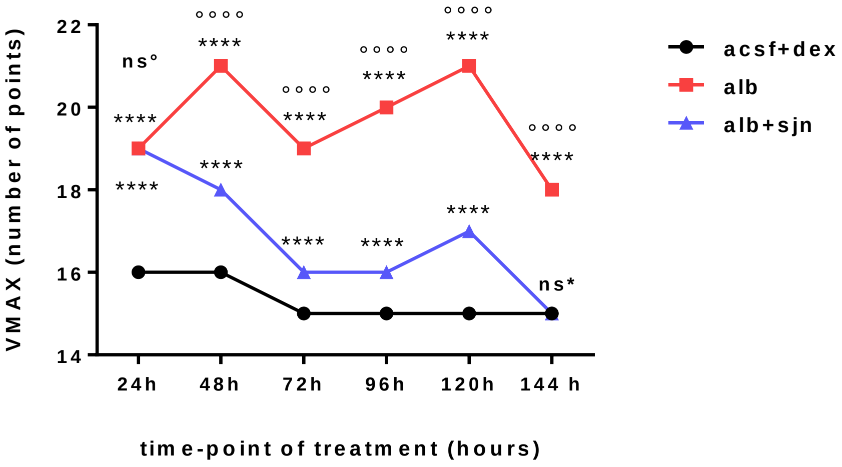

Legend of symbols: **** p<0.0001 vs. acsf+dex; °°°° p<0.0001 alb vs. alb+sjn; ns* not significant vs. acsf+dex; ns° not significant alb vs. alb+sjn

Supplementary Table S3. Detailed statistics of the variable TT

	Time-point of treatment (hours)					
	**24 h**	**48 h**	**72 h**	**96 h**	**120 h**	**144 h**
**Number of values**						
acsf+dex	2577	2522	2004	2594	2624	2678
alb	1870	1804	1332	1568	1299	673
alb+sjn	1631	1648	1253	1544	1274	974
**Median, percentile**						
acsf+dex						
25% Percentile	5.666	5.642	5.619	5.575	5.585	5.561
Median	5.934	5.922	5.913	5.86	5.833	5.842
75% Percentile	6.307	6.268	6.266	6.214	6.213	6.242
alb						
25% Percentile	5.834	5.834	5.777	5.838	5.879	5.702
Median	6.134	6.21	6.084	6.143	6.238	6.045
75% Percentile	6.489	6.667	6.598	6.603	6.651	6.46
alb+sjn						
25% Percentile	5.806	5.808	5.685	5.638	5.655	5.627
Median	6.199	6.143	5.943	5.874	5.895	5.975
75% Percentile	6.901	6.589	6.339	6.247	6.207	6.296
**Kruskal-Wallis test**						
P value	< 0.0001 ****	< 0.0001 ****	< 0.0001 ****	< 0.0001 ****	< 0.0001 ****	< 0.0001 ****
Kruskal-Wallis statistic	248.3	320.9	133.8	352.9	432.7	67.96
**Dunn's multiple comparisons test**						
acsf+dex Vs. alb - Adjusted P Value	< 0.0001 ****	< 0.0001 ****	< 0.0001 ****	< 0.0001 ****	< 0.0001 ****	< 0.0001 ****
acsf+dex Vs. alb+sjn - Adjusted P Value	< 0.0001 ****	< 0.0001 ****	0.0006 ***	0.0646 ns	0.0264 *	0.0084 **
alb Vs. alb+sjn - Adjusted P Value	0.0007 ***	0.0026 **	< 0.0001 ****	< 0.0001 ****	< 0.0001 ****	< 0.0001 ****

Legend: ns, not significant

Graphical representation of statistics of the variable TT, as reported in Supplementary Table S3

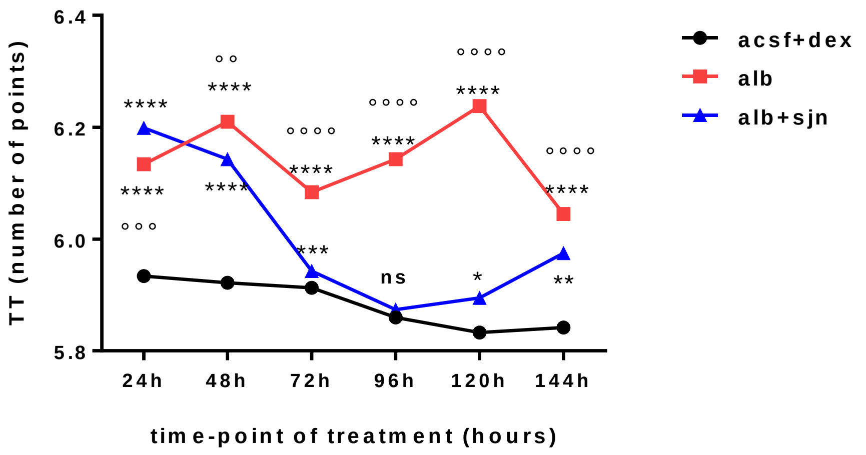

Legend of symbols: * p<0.05, ** p<0.01, *** p<0.001, **** p<0.0001 vs. acsf+dex; °° p<0.01, °°° p<0.001, °°°° p<0.0001 alb vs. alb+sjn; ns not significant vs. acsf+dex.

Supplementary Discussion

The RQA variables based on diagonal lines increase during epileptogenesis

The RQA also provided the measurements of the variables based on the diagonal lines of the recurrence plots, i.e., Determinism, Shannon Entropy and Longest Diagonal Line. These variables are defined as follows [5]:

- Determinism (DET), which represents the fraction of recurrence points forming diagonal lines. Diagonal lines represent epochs of similar time evolution of the states of the system. Therefore, DET is related with the determinism of the system and is expressed as

$$DET = \frac{\sum_{l=l_{min}}^{N} lP(l)}{\sum_{l=1}^{N} lP(l)} \qquad (5)$$

where l is the diagonal line length considered when its value is $\geq l_{min}$ and $P(l)$ is the probability distribution of the line lengths;

- Longest Diagonal Line (DMAX), which measures the dynamical stability of the system, being inversely related to the largest Lyapunov exponent

$$DMAX = max(\{l_i, i = 1, \ldots N_i\}) \qquad (6)$$

- Shannon Entropy (ENT) of the distribution of the line lengths, which is a measure of the complexity of the recurrence structure

$$ENT = -\sum_{l=l_{min}}^{N} p(l)\, ln\, p(l) \qquad (7)$$

where $p(l) = P(l)/N_l$ is the probability to find a diagonal line of exactly length l in the RP, being N_l the total number of diagonal lines.

Since the main finding is the emergence of the intermittency regime during the period of the epileptogenesis, we decided to report detailed statistics only for the RQA variables strictly correlated with this dynamical regime, and not to show statistics for the other variables. However, also the RQA variables based on the diagonal lines increase significantly during the period of the epileptogenesis.

Specifically, for the variable DET (which was normalized to the RAD, as for the variable LAM), the results are qualitatively and statistically similar to those of the variable LAM. This was expected, since the intermittency regime is characterized by the occurrence of bifurcations, that is, dynamic transitions of the system, which lead to a general increment of the occurrence of pseudo-periodic and/or slowly changing states, which increase the degree of predictability of the system, hence the increment of the variable DET. An analogous result was found during the period of epileptogenesis induced by SE [2]. Also the variables DMAX and ENT are significantly increased during the period of epileptogenesis, as expected also for these variables and similarly to what found during the period of epileptogenesis induced by SE [2]. However, during the first 96 hours of infusion of treatments, it is of interest to observe that for the experimental group of the animals treated with SJN2511, the measurements of the DMAX and the ENT are significantly higher than those met in the albumin treated group. Since the values of the variables LAM (fig. 1, panel A) and DET remain significantly higher than those measured in the control group, but lower respect to the albumin treated group, the increments of the variable DMAX and ENT are the expression of laminar/intermittency states. Nevertheless, the trajectories in the phase space of such laminar/intermittency states are denser, as reflected by the statistically significant decrease of the variable RAD, a phenomenon that occurs only for this specific group. This suggests a very different shaping of the trajectories in the phase spaces of the ECoG epochs selected from the SJN2511 treated group, maybe due to the occurrence of different types of intermittency, intermixed with

not identified transients dynamics. In authors' opinion, it cannot be excluded that the phenomenon of the statistically significant decrease of the variable RAD, followed by the increments of the variable DMAX and ENT, could represent the dynamic expression of the anti-epileptogenic mechanism(s) exerted by the SJN2511.

Laminar states occur also in physiological conditions

In authors' opinion, it is of interest to notice that also in the experimental control group, laminar states occur as expression of ongoing actual nonlinear phenomena, although at a weaker extent. This is supported by the validation test of our results (see the 'Methods' section, paragraph 'Validation test of results'), and suggests that the laminar/intermittency regime could be a dynamical state which may occur also without an overt pathology. Since 'micro-seizures' occur also in human healthy subjects [11], our findings lend support to the hypothesis that the main aberrant feature of the epileptic phenomena could be the spreading over abnormally extended spatio-temporal scales of physiological phenomena, which normally involve a reduced number of cells, for a short period of time [11, 12]. The occurrence of laminar states in basal conditions was detected also in our previous investigation [2].
